# Association of human cytomegalovirus (HCMV) neutralizing antibodies with antibodies to the HCMV glycoprotein complexes

**DOI:** 10.1186/s12985-020-01390-2

**Published:** 2020-08-03

**Authors:** Miho Shibamura, Tomoki Yoshikawa, Souichi Yamada, Takuya Inagaki, Phu Hoang Anh Nguyen, Hikaru Fujii, Shizuko Harada, Shuetsu Fukushi, Akira Oka, Masashi Mizuguchi, Masayuki Saijo

**Affiliations:** 1 Department of Virology 1, National Institute of Infectious Diseases, 1-23-1 Toyama, Shinjuku-ku, Tokyo, 162-8640 Japan; 2 Department of Pediatrics, Graduate School of Medicine, The University of Tokyo, 7-3-1 Hongo, Bunkyo-ku, Tokyo, Japan; 3 Department of Life Science and Medical Bioscience, Waseda University, 2-2 Wakamatsu-cho, Shinjuku-ku, Tokyo, Japan; 4 Department of Developmental Medical Sciences, Graduate School of Medicine, The University of Tokyo, 7-3-1 Hongo, Bunkyo-ku, Tokyo, Japan; 5 The Faculty of Veterinary Medicine, Okayama University of Science, Imabari, Ehime Japan

**Keywords:** Human cytomegalovirus, HCMV, Glycoprotein complex, Neutralization, Antibody

## Abstract

**Background:**

Human cytomegalovirus (HCMV) causes asymptomatic infections, but also causes congenital infections when women were infected with HCMV during pregnancy, and life-threatening diseases in immunocompromised patients. To better understand the mechanism of the neutralization activity against HCMV, the association of HCMV NT antibody titers was assessed with the antibody titers against each glycoprotein complex (gc) of HCMV.

**Methods:**

Sera collected from 78 healthy adult volunteers were used. HCMV Merlin strain and HCMV clinical isolate strain 1612 were used in the NT assay with the plaque reduction assay, in which both the MRC-5 fibroblasts cells and the RPE-1 epithelial cells were used. Glycoprotein complex of gB, gH/gL complexes (gH/gL/gO and gH/gL/UL128–131A [PC]) and gM/gN were selected as target glycoproteins. 293FT cells expressed with gB, gM/gN, gH/gL/gO, or PC, were prepared and used for the measurement of the antibody titers against each gc in an indirect immunofluorescence assay (IIFA). The correlation between the IIFA titers to each gc and the HCMV-NT titers was evaluated.

**Results:**

There were no significant correlations between gB-specific IIFA titers and the HCMV-NT titers in epithelial cells or between gM/gN complex-specific IIFA titers and the HCMV-NT titers. On the other hand, there was a statistically significant positive correlation between the IIFA titers to gH/gL complexes and HCMV-NT titers.

**Conclusions:**

The data suggest that the gH/gL complexes might be the major target to induce NT activity against HCMV.

## Introduction

Human cytomegalovirus (HCMV) is a member of the betaherpesvirinae sub-family of *Herpesviridae*, and is a cause of congenital transplacental infection of the fetus. Children with congenital HCMV infection have significant morbidity and mortality with symptoms that include permanent neurological defects, such as sensorineural deafness, developmental delay, dysopia, and epilepsy. HCMV also causes severe and sometimes lethal diseases in immunocompromised patients [1, 2].

HCMV glycoprotein (gP) complexes (gcs) expressed in the virion envelope function in the process of cell attachment and entry, and they are predominant targets of the virus-neutralization (NT) antibodies of HCMV. Among 23 gPs that are encoded in the HCMV genome, 9 gPs form 4 gcs: gB, gM/gN, gH/gL/gO and pentamer complex; gH/gL/pUL128/pUL130/pUL131A (PC), and all of these gcs are reported to have the potential to induce HCMV-NT antibodies [3–6].

Before the early 2000s, gB had been considered the main antigen in the development of the HCMV vaccine [7]. At that time, HCMV-NT activities were measured using fibroblast cells and the highly passaged laboratory HCMV strain, which lacked the expression of PC [8–11]. PC has recently been shown to be important for efficient entry of HCMV to epithelial cells (ECs), endothelial cells, and monocytes [12, 13], while the gH/gL/gO is required for the infection of both fibroblast cells and ECs [14]. Furthermore, it was reported that PC-specific antibodies might be a major component of HCMV-NT antibodies [6, 15–18].

For the development of an HCMV vaccine, the strong induction of NT activity against HCMV is desired. HCMV has many intricate evasion strategies against humoral immunity; these impede the development of an HCMV vaccine [19]. On the other hand, it is believed that potent HCMV-NT activity is required to protect a fetus from transplacental HCMV infection [20, 21]. Therefore, understanding the mechanism of HCMV NT antibody induction is necessary. We aimed to identify the antigen that actually induced the highest neutralizing activity by assessment for the correlation of anti-HCMV-NT antibody titers of healthy adult sera with the antibody titers against each of the following 4 gc antigens, gB, gM/gN, gH/gL/gO and PC.

## Materials and methods

### Cells

Fibroblast MRC-5 cells (American Type Culture Collection [ATCC® CCL-171]) were used. The MRC-5 cells were grown in Eagle’s minimum essential medium (MEM) supplemented with 10% heat-inactivated fetal bovine serum (Gibco, Carlsbad, CA), L-glutamine and sodium bicarbonate and 1% penicillin-streptomycin (MEM-10FBS). The retinal pigment epithelial cell line ARPE-19 (ATCC® CRL-2302™) and hTERT (human telomerase reverse transcriptase)-immortalized RPE-1 (ATCC® CRL-4000™), both of which were epithelial cell lines, were cultured in Dulbecco’s modified Eagle medium (DMEM)/F-12 (1:1), including L-glutamine and 2.438 g/L sodium bicarbonate (Gibco) supplemented with 10% heat-inactivated fetal bovine serum (Gibco or Hyclone, GE Healthcare UK Ltd., UK) (DMEM-10FBS). The culture medium for RPE-1 was additionally supplemented with hygromycin B at a concentration of 0.01 mg/mL. 293FT cells (Thermo Fisher Scientific, Waltham, MA) were grown in DMEM (Wako, Odawara, JAPAN) supplemented with 5% fetal bovine serum (Gibco) (DMEM-5FBS). The 293FT cells were cultured in collagen-coated plates (TOYOBO, Osaka, JAPAN).

### Viruses

HCMV strain Merlin (ME, ATCC® VR-1590™) was used as the source for constructing plasmids and for NT antibody analyses in MRC-5 cells. For the measurement of NT titers in ECs, HCMV clinical strain 1612, which was isolated in our laboratory from the urine of a 2-month-old baby with symptomatic HCMV disease was used. The HCMV 1612 strain was propagated in MRC-5 cells with three passages followed by five passages in ARPE-19 cells. In ARPE-19 cells infected with the 1612 strain, clear CPE was confirmed at 1 week after passage. The ME strain was mass-cultured following two passages in MRC-5 cells. The ME strain- or 1612 strain-infected cells were harvested at full CPE and suspended in FBS-free medium before being stored at − 80 °C. The stored cells were repeated freezing and thawing twice, and then the infectious dose in the supernatant was determined by counting CPE using MRC-5 cells. The titers of ME strain and 1612 strain measured using MRC-5 cells were 3.6 × 10^4^ plaque forming unit (PFU)/mL and 3.5 × 10^5^ PFU/mL, respectively. HCMV 1612 strain was used to measure the NT antibody titers in ECs, because the strain possessed the capacity to infect ECs.

### Serum sampling (subject selection) and ethical considerations

Seventy-eight healthy volunteers were recruited. The age of the volunteers ranged from 20 to 60 years (Table 1). All serum samples were first tested for HCMV IgG antibody positivity against total HCMV proteins with a commercially available enzyme-linked immunosorbent assay (ELISA) kit (DENKA SEIKEN, Tokyo, Japan), and the HCMV-IgG ELISA titers were determined according to the manufacturer’s instructions. The HCMV-IgG-positive sera were then further tested for HCMV-antibody titers by an indirect immunofluorescence assay (IIFA) and an HCMV-NT assay, as described below. The serum samples were heat-inactivated at 56 °C for 30 min before testing antibody titers with any assays.

**Table 1:** The HCMV-IgG antibody positive rate, as determined by HCMV-IgG ELISA

Age	HCMV-IgG positivity [No. of positive/No. of tested (%)]		
	Female	Male	Total
20–29	1/3 (33.3%)	2/4 (50%)	3/7 (42.9%)
30–39	10/15 (66.7%)	5/14 (33.3%)	15/29 (51.7%)
40–49	8/11 (72.7%)	11/15 (73.3%)	19/26 (73%)
50–59	3/6 (50%)	7/9 (77.8%)	10/15 (66.7%)
60–69	1/1 (100%)	0/0	1/1 (100%)
Total	23/42 (54.7%)	25/36 (69.4%)	48/78 (61.5%)

### HCMV neutralization assay

HCMV-NT titers of each serum were assessed with a conventional plaque reduction assay. In brief, serum samples were serially diluted with maintenance medium, MEM-2FBS or DMEM-2FBS. Sixty μL of the diluted serum sample and an equal volume of virus solution containing 60 plaque forming units (PFUs) of HCMV were mixed and incubated in a U-bottomed 96-well plate (Greiner Bio-One JAPAN, Tokyo, Japan) at 37 °C for 1 h. One hundred microliters of the mixture was then added to monolayers of ECs (RPE-1) or fibroblast (MRC-5) cells. The cell sheets were prepared by seeding cells to 15,000 cells per well in 96-well plates (CORNING, Corning, NY) on a day before performing the neutralization process. HCMV ME was used for the HCMV-NT assay in MRC-5 cells, while HCMV 1612 was used for the HCMV-NT assays in RPE-1 cells and MRC-5 cells. Fixation, staining with crystal violet, formalin and methanol treatment, and washing of the HCMV ME-MRC-5 plates were carried out at 4 days post inoculation, while the processing and measuring of NT antibody titers against HCMV 1612 in RPE-1 cells and MRC-5 cells were fixed at 2 days post inoculation. After staining, washing, and drying, CPE consisting of agglomerated cells were observed by stereomicroscopy. The 50% virus-NT titers (NT_50_) were defined as the reciprocal of the highest dilution level, at which the plaque number became less than half of the control. Each test was run in triplicate.

### HCMV glycoprotein genome amplification with polymerase chain reaction for plasmid construction

Each open reading frame (ORF) of the gP of HCMV ME was amplified by polymerase chain reaction (PCR) using primer sets that were designed with reference to the ME sequence (GenBank Accession no; AY446894.2.). All primers and oligonucleotides were purchased from Eurofins Genomics (Tokyo, Japan). The 30-μL reaction was composed of 15 μL Q5 High-Fidelity 2X Master Mix (New England Biolabs, Ipswich, MA), 0.5 μM of each primer, and template DNA. PCR amplicon bands were isolated from 10% agarose electrophoresis gel and were purified using the FastGene Gel/PCR Extraction Kit (NIPPON Genetics, Tokyo, JAPAN). Purified DNA was quantitated using a NanoDrop 2000c Spectrophotometer (Thermo Fisher Scientific).

### Plasmid construction

**Fig. 1:**
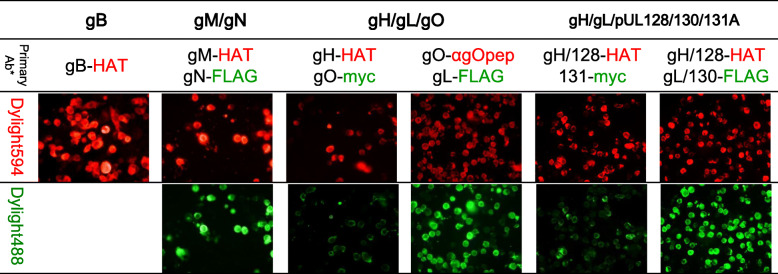
Confirmation of the expression of each membrane glycoprotein complex in 293FT cells transfected with each expression vector complex with an indirect immunofluorescence assay (IIFA) using antibodies to the tags fused with each membrane glycoprotein complex. The 293FT cells transfected with each designated plasmid or the combination of the plasmids were washed with phosphate buffered saline (PBS) (−), spotted on glass slides, and fixed and permeabilized with a methanol and acetone mixture. Glycoproteins that were expressed on each IIFA plate were described on the first line. Tag antibodies used as the primary antibodies are rabbit anti- HAT-IgG, mouse anti-FLAG, mouse myc-IgG, and rabbit anti-gO peptide antibody. The expression of Tag was distinguished by a secondary antibody (anti-rabbit IgG Dylight594 or anti-mouse IgG Dylight488). The tags fused with each gc were HAT (−gB, −gH, −gM, −pUL128), FLAG (−gL, −gN, −pUL130) and c-myc (−gO, −pUL131A)

First, the ORF of each gP of HCMV ME was amplified from cDNA, which was derived from RNA purified from the HCMV-ME-infecting MRC-5 cells by two step conventional reverse transcription. The synthetic DNA oligonucleotide of ME-UL128 wt (G > A in UL128 was fixed) was purchased from Integrated DNA Technologies (Coralville, IA). Each of the ORF genes was cloned into the cloning site of the modified pHEK293 ULTRA Expression vector II (Takara Bio Inc., Shiga, Japan), which was used for the recombinant protein expression in mammalian cells as a form of fusion protein with a designated tag at the carboxy-terminal (Fig. 1). The insertion of the gene of interest was carried out using Fusion™ HD (Takara Bio Inc) according to the manufacturer’s instructions.

### Sanger DNA sequencing

The nucleotide sequence was determined using an ABI Prism 3130 Avant Genetic Analyzer (Applied Biosystems, Foster City, CA). The sequences were aligned to the reference using DNA Dynamo (Blue-Tractor Software, North Wales, UK). The nucleotide sequence of the constructed plasmids was confirmed to be the original sequence by Sanger DNA sequencing.

The sequence of the UL128L (UL128–131A locus) in strain 1612 was determined by Sanger sequencing, in which the PCR product was amplified using primers UL128L-F (GCGTATTTCGGACAAACACACA) and UL128L-R (CGCATGTTGCAGACTGAGAAAGA) [22]. It was confirmed that there were no mutations in the UL128L gene of the HCMV 1612.

### Antigen preparations for the indirect immunofluorescence assay

293FT cells were transfected with pHEK293-gB for the expression of recombinant gB. The same cells were also co-transfected with pHEK-gM and pHEK-gN, with pHEK-gH, pHEK-gL, and pHEK-gO, and with pHEK-gH, pHEK-gL, pUL128, pUL130, and pUL131A for the expression of gM/gN, gH/gL/gO, and PC, respectively, using pHEK293 Enhancer Vector (Takara Bio Inc.) and HuGENE HD (Promega).

The 293FT cells transfected with each designated plasmid or the combination of the plasmids were washed with phosphate buffered saline (PBS) (−), spotted on glass slides (Matsunami Glass IND., Ltd., Osaka, Japan), and fixed with a methanol and acetone mixture mixed at a ratio of 1:1.

The expression of gc was confirmed by IIFA by detecting the respective tags. The antibodies, which were used for the detection of histidine affinity *tag* (HAT)-fusion protein, c-myc-fusion protein, and FLAG-tag fusion protein, were rabbit anti-HAT-tag polyclonal antibody (GenScript, Piscataway, NJ), mouse MYC-monoclonal antibody (Aviva Systems Biology, San Diego, CA), and anti-FLAG M2 monoclonal antibody (Sigma-Aldrich Japan, Tokyo, Japan), respectively. The secondary antibodies were Alexa Fluor DyLight 488-conjugated goat anti-mouse IgG H + L antibody or DyLight 594-conjugated goat anti-rabbit IgG H + L antibody (Invitrogen). Anti-gO peptide rabbit antibodies (peptide sequence: KLKRKQALVKEQPQKKNKKS [23]) were produced by Eurofins Genomics (Tokyo, Japan).

### Detection of antibodies to each gc in indirect immunofluorescence assay

To measure each gc-specific IIFA titer in sera, the serum samples were two-fold serially diluted with PBS and added onto the glass slides. After incubation at 37 °C for 1 h, the cells were washed with PBS 3 times, and were then reacted with fluorescein isothiocyanate (FITC)-conjugated goat anti-human IgG H + L (Invitrogen, Carlsbad, CA). The antibody titer was defined as the reciprocal of the highest dilution level, at which the specific fluorescent signal was detected. The cells transfected with plasmid vectors without each gp insert were used as negative control. Two samples of CMV-IgG-negative sera were tested negative for antibody detection in IIFA. Since there was a concern about subjectivity with regard to the measurement of specific antibody titers in IIFA, the FITC-specific signal was observed by two experts to ensure consistency. To avoid detecting artifacts as much as possible, the signals in the fixed cells were observed at a lower magnification first, and they were again observed at a higher magnification. Clearly positive signals, which were different from those of the negative control, were determined positive.

### Statistical analysis

Statistical analyses were performed using the Stata15 software program (STATA Corporation, College Station, TX). Non-parametric analyses of the correlations were performed using Spearman’s test. *P* values of < 0.05 were considered to indicate statistical significance.

### Next generation sequencing

Amino-acid sequence homology of HCMV ME and HCMV 1612 was confirmed following base sequence determination using a next generation sequencer (NGS). Genomic DNA of HCMV 1612 was extracted from ARPE-19 cells infected with HCMV 1612 using a QIAmp DNA Mini Kit (QIAGEN, Hilden, Germany) after repeated freeze-thaw cycle treatment 2 times. The sequencing libraries were then prepared by an Ion Xpress Plus Fragment Library Kit (Thermo Fisher Scientific) in accordance with the manufacturer’s instructions. The library concentration was quantified using the Ion Library TaqMan Quantification Kit (Thermo Fisher Scientific). An emulsion PCR was performed on the library, which was adjusted to a 50-pM concentration, pooled in equimolar amounts, and mixed with capture beads on the Ion Chef System (Thermo Fisher Scientific) supplemented with the Ion Torrent Personal Genome Machine (PGM) template 200 kit (Thermo Fisher Scientific). The template libraries were sequenced with the Ion Torrent PGM using the Ion 314 Chip Kit v2 (Thermo Fisher Scientific) and the Ion Torrent PGM Sequencing 200 Kit v2 (Thermo Fisher Scientific), according to the manufacturer’s instructions. The resulting FASTQ format files were imported into CLC Genomics Workbench 9.0.1 (QIAGEN) for a homology analysis. Each gP sequence of 1612 strains were registered in GenBank with the accession numbers LC425070-LC425078.

## Results

### Confirmation of the gc expression for the detection of antibodies to IIFA antigen

The expression of all 4 types of gc was confirmed by IIFA through the detection of each of the fused tags (Fig. 1). The tag fused with gB, gH, gM, and pUL128 was HAT, while that with gL, gN, pUL130 was FLAG (−) and that with gO and pUL131A was c-myc. The tags assigned to the gp comprising each complex were differentiated by using two different fluorescent labels of the secondary antibodies. It was confirmed that the expression of each gp except for gO with Western blotting (data not shown). The expression of gO, which was fused with several different tags at difference position (C-terminus or N-terminus) was examined with WB and IIFA using anti-gO peptide antibodies or the anti-designated tag antibodies repeatedly, but no signals for gO expression were detected.

### HCMV IgG prevalence among volunteers

The HCMV ELISA-IgG prevalence rate determined in the ELISA is shown in Table 1. The overall positive rate was 61.5%. After whole population was divided into two groups, one under 39 years of age and the other over 40 years of age. The prevalence of HCMV-IgG antibody positive rate among those aged equal to or over 40 was significantly higher among those aged less than 40 (*p* = 0.02, Student’s t-test). There was no significant difference in the prevalence of IgG between men and women.

### Correlation between the HCMV ELISA-IgG titers and the HCMV-NT antibody titers

**Fig. 2:**
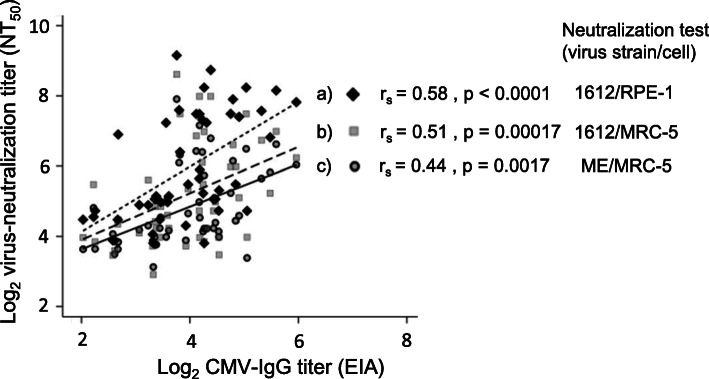
Correlation between the quantitative ELISA HCMV-IgG titers and the HCMV-NT titers (NT_50_). The correlations were analyzed using Spearman’s test. The strength of the correlation was expressed using the correlation coefficient “r_s_”. *P* values of < 0.05 were considered to indicate statistical significance. The r_s_ and *p* values were 0.58 (**a**; closed diamond), 0.51 (**b**; square) and 0.44 (**c**; closed circle) for HCMV 1612/cell RPE-1 (**a**), HCMV 1612/cell MRC-5 (**b**), and HCMV ME/cell MRC-5 (**c**) combinations, respectively

Correlation between the ELISA HCMV-IgG titers and the NT_50_ titers was assessed using Spearman’s test. The correlation coefficient “r_s_” were 0.58, 0.51 and 0.44 in NT assay using HCMV 1612/RPE-1 cells, HCMV 1612/MRC-5 cells, and HCMV ME/MRC-5 cells combinations, respectively. Thus, there was a relatively moderate statistically significant correlation between the HCMV ELISA-IgG titers and the HCMV-NT titers, regardless of the combinations between cells and virus types (Fig. 2). All *P*-value were less than 0.01.

### Relevance of using HCMV strain 1612

**Fig. 3:**
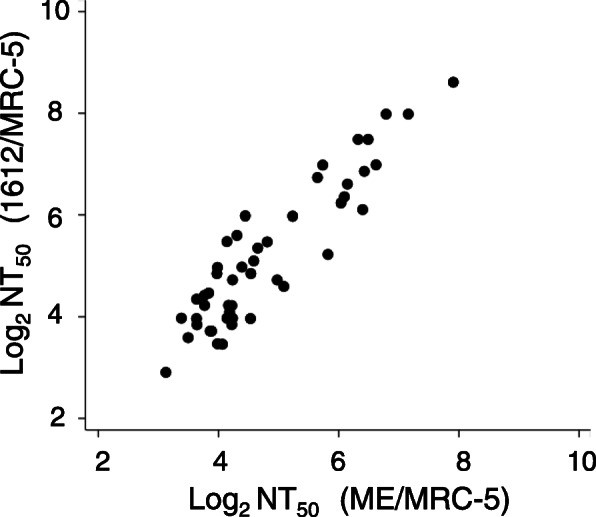
The correlation between the NT antibody titers of 78 participants determined using HCMV ME in MRC-5 fibroblast cells and those determined using HCMV 1612 in MRC-5 fibroblast cells. A strong correlation was observed with an r_s_ value of 0.85 (*p* < 0.05)

The amino acid sequence homology of gB, gH, gL, gO, gM, gN, pUL130, and pUL131A between HCMV1612 and HCMV ME was 99.4, 99.2, 98.9, 99.6, 100, 99.3, 98.6, and 100%, respectively. The amino acid sequence homology of pUL128 between strains 1612 and ME wild-type (Merlin BAC in which UL128 is repaired [21, 24]) was 99.4%. In addition to the amino acid homology, we also evaluated the phenotypic homology of strain ME with strain 1612 (i.e., the NT antibody titers to each strain determined in MRC-5 cells were compared). There was a positive correlation between the NT_50_ determined in fibroblasts against HCMV ME and those against HCMV 1612 (Fig. 3), indicating the validity of using the clinical isolate HCMV 1612.

### Correlation between NT_50_ and gc specific antibody titers determined with IIFA

**Fig. 4:**
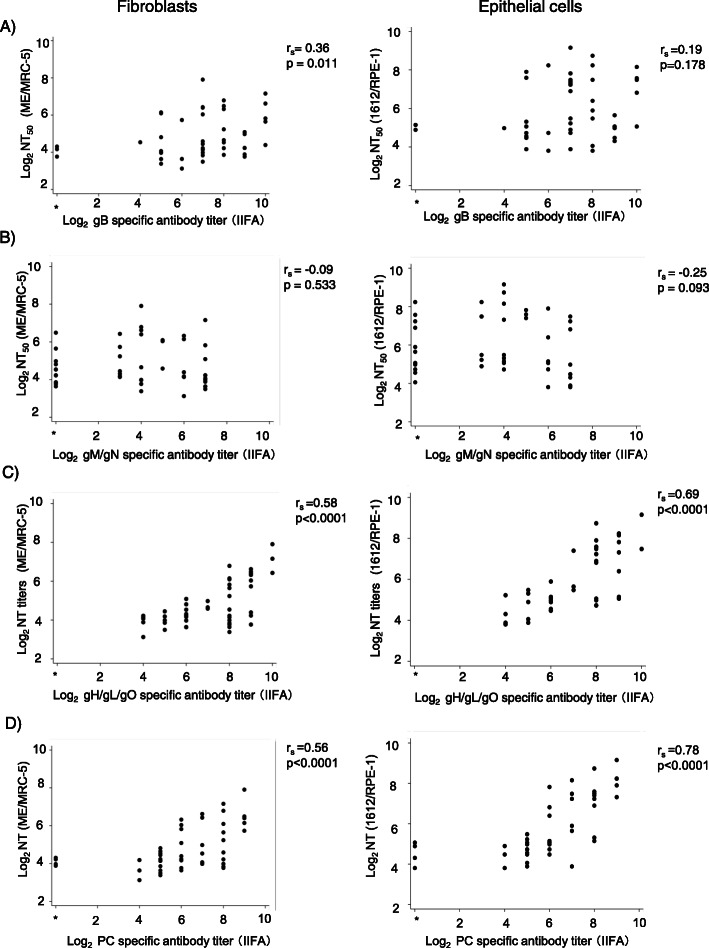
The correlation of each anti-gc specific IIFA titer with HCMV-NT_50_ titers. The HCMV strain and cell types used in the neutralization test were HCMV ME/MRC-5 fibroblasts (left panels of **a**, **b**, **c**, and **d**), and HCMV 1612/RPE-1 epithelial cells (right panels of **a**, **b**, **c**, and **d**). Each correlation coefficient r_s_ and *p*-value is indicated in the figure. “Negative” in IIFA means a result of < 8 (1 × 2^3^). All samples were HCMV-NT_50_-positive (> 8) and showed a positive CMV-IgG titer (EIA). *The correlation coefficient was calculated regarding IIFA-negative as 1, that is indicated as 1 × 2^0^

The correlation between the HCMV-NT_50_ and each gc-specific IIFA antibody titers is shown in Fig. 4. gB-specific IIFA antibody titers showed a statistically significant positive correlation with the HCMV-NT_50_ titers to both HCMV ME and 1612 determined in fibroblast cells (Spearman R = 0.36, *p* = 0.011), but not with the HCMV-NT_50_ to 1612 strain determined in ECs (R = 0.19, *p* = 0.17). Anti-gM/gN IIFA antibody titers did not show any correlation with the HCMV-NT_50_ in fibroblasts or ECs (r_s_ = − 0.09 and *p* = 0.533 in fibroblast cells; r_s_ = − 0.25 and *p* = 0.092 in ECs) (Fig. 4b). In contrast, gH/gL complex-specific IIFA titers had a statistically significant correlation with HCMV-NT_50_ titers (Fig. 4c and d), as a correlation analysis, gave Spearman’s R values of 0.58, 0.69, 0.56, and 0.78 for gHLO/NT (Fibroblasts), gHLO/NT (ECs), PC/NT (Fibroblasts), and PC/NT (ECs), respectively; all *p* values were < 0.05.The highest correlation was demonstrated between the HCMV-NT_50_s determined in ECs and PC-specific IIFA titers (R = 0.78, *p* < 0.0001, Fig. 4d, right panel). Both gH/gL/gO-specific IIFA antibody titers and PC-specific IIFA titers showed a higher correlation with the HCMV-NT_50_s determined in ECs in comparison to those determined in fibroblasts. There was also a statistically significant positive correlation between the gH/gL/gO-specific IIFA titers and the PC-specific IIFA titers (R = 0.5967, *p* < 0.001).

## Discussion

The HCMV IgG seroprevalence rate of subjects (approximately 60%) in the present study is compatible with previously reported data in Japan [25, 26]. The HCMV-NT_50_ titers showed a weak to moderate correlation with EIA IgG-antibody titers in either virus–cell combination (Fig. 2), as reported previously [27–29]. When pregnant women are infected with HCMV as the primary infection, the IgG antibody, as determined by the EIA, become positive. The levels are parallel to the NT-antibody titers, and the antibody level peaked at approximately 3 months after infection, and are maintained for approximately 1 year [18]. The present study demonstrated a moderate correlation between NT-antibody titers and EIA-HCMV IgG titers.

This study confirmed the expression of each gP, with the exception of gO, by Western blotting (data not shown). The single expression of gO was not observed by any methods (Fig. 2). The gH/gL complex is stabilized as a complex form by binding with gO or UL128, UL130 and UL131A; thus, tags and peptide antigens were considered to be exposed after conformational changing of the expressed protein [30, 31].

In the last 20 years, it has been recognized that gB might be the main target with which NT antibodies react [7]. Originally, the development of gB-based vaccines has been driven by the observation that adsorption treatment of human sera with using recombinant gB diminishes HCMV entry to fibroblast cells through neutralizing activity by approximately 50% [4, 32–34]. It was also reported that gB-specific antibody titers were correlated with NT_50_ titers [35]. This evidence was obtained from experiments in which the serum NT activity was explored using fibroblasts (mainly, human embryo lung fibroblast cells) and highly-passaged laboratory HCMV strains, such as the HCMV Town strain. As HCMV-NT_50_ titers differ greatly depending on the type of cells and HCMV strains used, we measured the HCMV-NT_50_ titers using 3 types of HCMV-cells combination as described above. It was revealed that there was a positive correlation between gB-specific IIFA titers and the HCMV-NT_50_ titers when the HCMV-NT_50_ was determined in MRC-5 fibroblast cells but not in RPE-1 ECs (Fig. 4a), suggesting that the correlation is dependent on the virus strain and cells used for measuring HCMV-NT antibodies. In addition, it was revealed that antigenic target sites, with which NT antibodies react, were more heavily glycosylated than those that elicit non-NT antibodies, suggesting that HCMV-gB shields NT epitopes by taking advantage of glycosylation [36]. It has also been shown that the partial effect of the gB subunit vaccine is independent on NT antibody induction [37, 38], and that the vast majority (> 90%) of gB-specific antibodies secreted from B-cell clones do not have NT activity against HCMV [39]. Furthermore, adsorption studies with recombinant gB in sera from naturally infected humans suggest that anti-gB antibodies do not contribute significantly to HCMV EC entry through neutralizing responses [15, 17]. These reports support the results obtained in the present study that anti-gB specific IIFA titers in sera showed little correlation with the NT antibody titers determined in the HCMV 1612/RPE-1 ECs combination. However, it should be note that neutralizing assays were conducted in the absence of complement. Given that at least two neutralizing epitopes in gB have been shown to be fully dependent on complement, that neutralizing activities induced by gB-subunit vaccines often exhibit profound complement dependence, and that fibroblast neutralizing activity of human convalescent sera was 2 to 4 fold enhanced by the presence of complement [40, 41]. It would be important to note that the contribution made by gB-specific antibodies to fibroblast neutralizing activities may have been under-represented because endogenous complement in the sera was heat inactivated and exogenous complement was not added after heat inactivation. Consequently, the correlation between gB antibodies and neutralizing activities might be stronger if neutralizing assays were conducted in the presence of complement. It was reported that the structural and antigenic analyses showed that the postfusion gB trimer presented complement-independent neutralizing sites [42]. Thus, modifications on the gB trimer surface that reduce exposure to non-neutralizing sites, such as avoidance of glycosylation, may result in an improvement of HCMV vaccine development.

Although the function of gM/gN is largely unknown, there are a few previous reports that indicate gM/gN can elicit a virus-neutralizing antibody response [5, 43]. However, no correlation was found between anti-gM/gN IIFA antibody titers and NT_50_ titers (Fig. 4b). It was reported that immune evasion from NT antibodies became possible by the glycosylation of gN [44]. The result that the IIFA gM/gN-IgG titers were not significantly correlated with the HCMV-NT_50_ titers in this study might be due to the glycosylation of gN. Because the gM/gN is the glycoprotein complex that is the most abundantly expressed on viral particles [45], glycosylation of gM/gN could reduce the NT effect by NT antibodies, as in the case of gB [36]. Further studies are needed to reveal the mechanism of host immune evasion by gM/gN, if gM/gN could induce the immune evasion. Furthermore, it should be clarified whether gM/gN complex has an ability to induce HCMV neutralizing antibodies.

Of all the combinations of cells and gc that were examined in this study for the correlation between NT_50_ titers and specific IIFA antibody titers against gc, the strongest correlation was found between anti-PC IIFA antibody titers and NT_50_ titers measured in ECs (Fig. 4d). Furthermore, the anti-gH/gL/gO IIFA titers were also significantly correlated with the HCMV-NT_50_ titers (Fig. 4c). These glycoprotein complexes seem to be the most important antigen in the induction of NT antibodies, as reported previously [17, 30, 46–49]. In addition, there are multiple neutralizing epitopes in gH/gL/gO. Those that are defined only by gH/gL sequences are common in both gH/gL/gO and the pentameric complex and for the most part block both fibroblast and epithelial cell entry, while those that are defined by UL128/UL130/UL131A sequences only block epithelial cell entry. There are epitopes on gH/gL within PC that probably associated with HCMV entry to fibroblast cells. Thus, the presence of the common epitopes is likely to have a major impact on the positive and significant correlation of both anti-PC and anti-gH/gL/gO IIFA antibody titers with HCMV-NT_50_ titer (Fig. 4c and d) [31].

One limitation of our study is that we used fixed and permeabilized cells for IIFA. Thus, as serum antibodies would have access to the viral proteins expressed not only on the virion surface but also inside the cells, suggesting the IIFA positive signals might be due to the antibodies that reacted with individual gc, misfolded gc, or partial degradation products of gc. In contrast, IIFAs based on non-permeabilized cells might reveal more robust correlations with neutralizing activities, as prior studies suggest that cell surface expression of both gH/gL and gM/gN complexes is restricted to conformationally native complexes [30, 50–53]. Thus, the antibodies, which react with these complexes in their native and quaternary conformations, would be selectively detected with IIFA, in which non-permeabilized cells were used for IIFA antigens. Therefore, further studies are needed to eliminate IIFA signals due to the antibodies react with non-native epitopes, which presumably do not exhibit neutralizing activity.

To the best our knowledge, because there have not been reported similar studies that included gO in the gH/gL complex or gM/gN. the results are well worth reporting. The findings will be of particular importance in the vaccine and immunotherapeutics development against HCMV infections.

## Conclusion

Both the anti-PC antibody titers and the anti-gH/gL/gO antibody titers determined in the IIFA were highly correlated with the HCMV-NT_50_ titers determined in both HCMV ME-fibroblasts and HCMV 1612-epithelial cell combinations. By measuring the antibody titers to gH/gL/gO or PC in IIFA, it is possible to estimate the level of the HCMV-NT_50_ titer. The data obtained in this study suggest that the induction of a strong immune response to gH/gL complexes is important for the development of HCMV vaccines, which might have an ability to reduce the risk of congenital HCMV infection.

## Data Availability

The datasets used and/or analyzed during the current study are available from the corresponding author on reasonable request.

## Abbreviations

DMEM: Dulbecco’s modified Eagle medium
EC: Epithelial cells
ELISA: Enzyme-linked immunosorbent assay
FITC: Fluorescein isothiocyanate
gc: Glycoprotein complex
gP: Glycoprotein
HCMV: Human cytomegalovirus
IIFA: Indirect immunofluorescence assay
NGS: Next generation sequencer
NT: Neutralization
NT_50_: Fifty percent virus-NT titers
ORF: Open reading frame
PC: Pentamer complex
PCR: Polymerase chain reaction
PFU: Plaque forming unit
PGM: Personal Genome Machine
